# Huaier Extract Inhibits Prostate Cancer Growth *via* Targeting AR/AR-V7 Pathway

**DOI:** 10.3389/fonc.2021.615568

**Published:** 2021-02-23

**Authors:** Zhengfang Liu, Cheng Liu, Keqiang Yan, Jikai Liu, Zhiqing Fang, Yidong Fan

**Affiliations:** ^1^ Department of Urology, Qilu Hospital of Shandong University, Ji’nan, China; ^2^ Department of Urology, Peking University Third Hospital, Beijing, China; ^3^ Department of Medicine, Center for Molecular Medicine (CMM) and Bioclinicum, Karolinska Institute and Karolinska University Hospital Solna, Solna, Sweden

**Keywords:** huaier extract, prostate cancer, full length androgen receptor (AR-FL), androgen receptor splicing variant 7 (AR-V7), enzalutamide

## Abstract

The androgen receptor (AR) plays a pivotal role in prostatic carcinogenesis, and it also affects the transition from hormone sensitive prostate cancer (HSPC) to castration-resistant prostate cancer (CRPC). Particularly, the persistent activation of the androgen receptor and the appearance of androgen receptor splicing variant 7 (AR-V7), could partly explain the failure of androgen deprivation therapy (ADT). In the present study, we reported that huaier extract, derived from officinal fungi, has potent antiproliferative effects in both HSPC and CRPC cells. Mechanistically, huaier extract downregulated both full length AR (AR-FL) and AR-V7 mRNA levels *via* targeting the SET and MYND domain-containing protein 3 (SMYD3) signaling pathway. Huaier extract also enhanced proteasome-mediated protein degradation of AR-FL and AR-V7 by downregulating proteasome-associated deubiquitinase ubiquitin-specific protease 14 (USP14). Furthermore, huaier extract inhibited AR-FL/AR-V7 transcriptional activity and their nuclear translocation. More importantly, our data demonstrated that huaier extract could re-sensitize enzalutamide-resistant prostate cancer cells to enzalutamide treatment *in vitro* and *in vivo* models. Our work revealed that huaier extract could be effective for treatment of prostate cancer either as monotherapy or in combination with enzalutamide.

## Introduction

With nearly 1,276,106 new cases expected worldwide in 2018, prostate cancer (PCa) represents the second-most frequent type of cancer in men following lung cancer, accounting for almost 7.1% of new cancer cases, and there were 358,989 prostate cancer–related deaths in 2018 around the world according to the GLOBOCAN 2018 (1). Unfortunately, some cases are in advanced stages at the time of their initial diagnosis, particularly in unscreened populations, of which ≥10% appear with metastatic disease at first presentation (2).

The androgen receptor (AR), consisting of four domains with 919 amino acids in full-length, is the core of cellularly physiological and pathophysiological process in prostate cell (3–7). The activity of AR needs the stimulation of ligand (e.g., dihydrotestosterone), after activated, it translocates to the nucleus and interacts with promoters of downstream genes to promote their transcriptions (8, 9). Therefore, androgen deprivation therapy (ADT) is the basic therapy for PCa. Unfortunately, majority of cases will progress to the more aggressive stages, or castration-resistant prostate cancer (CRPC), within 3 years, and the average overall survival for metastatic CRPC patients is about 1.5 years (9–11).

Recently, enzalutamide, the second-generation of hormonal drug targeting full-length AR (AR-FL), were used to treat CRPCs (12). Nevertheless, majority of patients progress shortly after enzalutamide treatment (13–15). Ligand independent signaling and gene amplification of AR contribute to the enzalutamide resistance, especially the emergence of androgen receptor splice variants (16). Androgen receptor variants (AR-Vs) maintain the transcription domain and promote target genes activity not dependent on ligand binding (17–19). Among the many androgen variants, AR-V7, which is in absence of ligand-binding domain, and only retains the key domain for functional activation, the N-terminal domain and DNA-binding domain, has received the most attention, as it has guiding significance for doctors to management of prostate cancer (20–23). Unfortunately, there is no drug especially targeting AR-V7, and this situation presents huge challenges for PCa therapy. Thus, targeting the AR axis, especially AR-V7, could be a promising treatment for PCa.

*Trametes robiniophila murr* (huaier), a type of medicinal fungus which is found on the trunks of *Styphnolobium japonicum* (distributed across Hebei, Shandong, and Shanxi Provinces, China), has been used in Traditional Chinese Medicine (TCM) for approximately 1,600 years (24–26). Nowadays, huaier granule is a Chinese State Food and Drug Administration (SFDA)–approved drug used in the clinic. Its proteoglycans (protein-bound polysaccharides) have been proven to be the active ingredient in huaier extract (**Supplementary Table 1**). Recent studies reveal that huaier extract can act as an efficacious tumor suppressor for a range of tumors (25, 26). Huaier extract antitumor potential are mainly involved in antiproliferation, anti-metastasis, tumor-specific immunomodulatory, and cancer stem cell inhibitory activities, and these effects attribute to regulate many onco-driving signaling pathways, such as Wnt/β-catenin pathway, NF-κB pathway, AKT/mTOR pathway, and p53 pathways (27). Although recent study revealed its antitumor activity in breast cancer cell by modulating the ERα pathway (27), the studies of huaier on prostate cancer are rare. To our knowledge, there is no study on huaier extract targeting the AR/AR-V7 signaling pathway in PCa.

Here, we demonstrate that huaier extract acts as potent AR-FL and AR-V7 inhibitors in PCa. It can inhibit PCa cell viability *in vitro* and *in vivo* by targeting both AR-FL and AR-V7 signaling pathways. Furthermore, huaier extract shows synergistic effects in combination with enzalutamide and enhances enzalutamide therapy in PCa. In conclusion, huaier extract may act as a promising drug candidate for treatment of PCa.

## Materials and Methods

### Cell Culture and Reagents

LNCaP, 22Rv1, and PC3 prostate cancer cells were purchased from Cell Bank of Chinese Academy of Sciences (Shanghai, China). Cell lines were routinely tested mycoplasma free and were authenticated by STR detection. The cell lines were cultured in RPMI1640 with 10% fetal bovine serum (FBS), and the culture media for 22Rv1 also contained 1× GlutaMAX™ (Invitrogen, Carlsbad, CA, USA), and 1mmol/L Sodium Pyruvate (Invitrogen, Carlsbad, CA, USA). Enzalutamide, MG132, and IU1 were purchased from MedChemExpress (Monmouth Junction, NJ, USA). Huaier extract was kindly provided by Gaitianli Medicine Co., Ltd. (Qidong, Jiangsu, China), and was prepared as previously described (24, 25).

### Cell Viability Assay

Cell proliferation was assessed by the cell counting kit 8 (CCK-8) assay according to the manufacturer’s Instruction (Dojindo Molecular Technologies, Shanghai, China). Briefly, PCa cells (4 × 10^3^ cells/well) in 100 μl of medium were seeded in 96-well plates. After 12-h culture in 5% CO2 at 37°C in a humidified incubator, the medium was replaced by different concentrated solutions of huaier extract, and then incubated for another 24–72 h. Afterward, 10 μl CCK-8 was added to each well and the cells were incubated for another 1–4 h at 37°C according to the instructions of the manufacture. Absorbance of each well was quantified at 450 nm by an enzyme-linked immunosorbent assay microplate reader (Tecan Trading AG, Switzerland).

### Colony Formation Assay

LNCaP and 22Rv1 cells were seeded in 100-mm dishes at 1 × 10^4^ cells/dish with different concentration of huaier extract. After 14 days, cells were washed with PBS twice, fixed with 4% paraformaldehyde, and stained with 0.1% crystal violet.

### Invasion Assay

The Transwell system (Corning Costar, Lowell, USA) were used in invasion assay. In addition, 2 × 10^5^ LNCaP and 22Rv1 cells, suspended in 100-ul serum-free medium with/without 2 mg/ml huaier extract, were seeded in the upper chambers with the basement membrane, and 500-ul medium containing 10% FBS was added to the bottom chamber. After 48 h, the invaded cells were stained with 0.1% crystal violet and the images of invaded cells were acquired using the microscope (Leica Biosystems Nussloch GmbH, Germany).

### Migration Assay

The migration assay was performed in the same way as the invasion assay except the following differences: no Matrigel using 1 × 10^5^ cells/well seeded, 36-h incubation.

### Flow Cytometry Analysis of Apoptosis

PCa cells were treated with huaier extract for 48 h, and the cells were collected in 100-ul binding buffer and incubated with PI and Annexin V-fluorescein isothiocyanate (BestBio, Shanghai, China) in the dark for 15 min at room temperature. Then 400-ul binding buffer was added to each tube, and fluorescence activated cell sorting (FACS) was performed immediately.

### Flow Cytometry Analysis of Cell Cycle

Cell-cycle analysis was performed using a Cell Cycle Assay Kit Plus (US Everbright, Suzhou, Jiangsu, China) according to the manufacturer’s instructions. Briefly, PCa cells were treated with huaier extract for 48 h, and the cells were collected, washed with cold PBS twice and fixed with 75% ethanol at 4°C overnight. Then the cells were wash with PBS, and suspended in 1ml PBS, followed by the addition of 4-ul RedNucleus I (far infrared nucleic acid dye, excited at 638 nm) for 20 min in dark at room temperature. Fluorescence activated cell sorting (FACS) was performed at channel FL4 immediately.

### siRNA and Plasmids Transfection of Cells

Cells were seeded in six-well plates and transfected with small-interfering RNA (siRNA) (GenePharma, Shanghai, China; Santa Cruz Biotechnology, Santa Cruz, CA, USA) using jetPRIME (polyplus-transfection, Illkirch, France). To overexpress target genes in prostate cancer cells, pGL3-basic vector containing target genes cDNAs (genechem, Shanghai, China) transiently transfected the PCa cells *via* jetPRIME.

### Luciferase Reporter Assay

For luciferase reporter assay, PC3 cells (2 × 105 cells per well of six-well plate) were transfected with pGL3-KLK3-Luc reporter plasmid or the control plasmid along with AR-FL or AR-V7. The luciferase activity was determined 24 to 48 h after transfection using a dual luciferase reporter assay system following the manufacturer’s instructions (Promega, Madison, WI, USA), the signal was normalized to Renilla luciferase control as relative luciferase units.

### Immunofluorescence Assay

PC3 cells were transfected with vector containing AR-FL cDNAs (genechem, Shanghai, China), and AR-V7 cDNAs (genechem, Shanghai, China) *via* jetPRIME (polyplus-transfection, Illkirch, France). After 24 h, cells were treated with either huaier extract (2 mg/ml) or enzalutamide (20 umol/L) for another 24 h and subsequently treated with dihydrotestosterone (DHT) (10 nmol/L) for 2 h. Cells were then fixed with Paraformaldehyde, and permeabilized with 0.4% Triton X-100. After blocking by goat serum for 1 h, cells were incubated with anti-AR-FL (Cell Signal Technology, Danvers, MA, USA) and anti-AR-V7 antibodies (Cell Signal Technology, Danvers, MA, USA) overnight at 4°C. The slides were then incubated in CoraLite488–conjugated affinipure goat anti-rabbit IgG (proteintech, Wuhan, Hubei, China) for 1 h. The images were obtained by the Olympus microscope equipped with PerkinElmer system.

### Co-Immunoprecipitation and Western Blot

For immunoprecipitation (IP), the cell lysates, prepared with agarose protein A+G (Santa cruz biotechnology, Dallas, Texas, USA) to eliminate nonspecific binding, were incubated with primary antibody overnight at 4°C, and mixed with agarose protein A+G for 6 h at 4°C. After washed three times by lysis buffer, the loading buffer was added and the agarose protein A+G were removed by centrifugation. Western blot was performed to analyze protein expression. For western blot, it was performed according to the standard method, briefly, 30 ug total protein was loaded on gel, after the progress of running the gel, transferring the protein, and incubating with antibodies, the protein image was acquired by the chemiluminescence detection system.

### Real-Time Quantitative Polymerase Chain Reaction

RNAs were extracted using Trizol (TaKaRa Bio, Dalin, China). One microgram of total RNA was used for cDNA synthesis by the SuperScript III Reverse Transcriptase Kit (TaKaRa Bio, Dalin, China). For detection of the indicated genes, each cDNA sample was amplified using SYBR Green (TaKaRa Bio, Dalin, China) *via* QuantStudio 3 Real-Time PCR system (Thermo Fisher Scientific, Waltham, MA, USA). Primers used for PCR were showed in **Supplementary Table 2**.

### Immunohistochemistry

The formalin fixed tissues were paraffin-embedded and sliced into 4-um slides. After deparaffinization, hydration, and antigen retrieval, blocking endogenous peroxidase and nonspecific antibody binding were carried out followed by incubation with primary antibody. DAB developing were carried out after the slides were incubated with secondary antibody labeled with HRP. Finally, the slides were counterstained with hematoxylin, dehydrated with ethanol, cleaned with xylene, and mounted with resin mounting medium. Images were taken with CIC XSP-C204 microscopy.

### *In Vivo* Tumorigenesis Assay

The animal experiments were performed in strict accordance with the Guidelines for the Care and Use of Laboratory Animals of Shandong University. Five- to six-week-old male BALB/c-nu mice were purchased from Charles River Laboratories. 5 × 10^6^ 22Rv1 cells were subcutaneously injected into the flanks of the mice. When tumor volume reached 50–100 mm^3^, mice were randomized into four groups and treated as follows: (i) vehicle control (normal saline, p.o.), (ii) enzalutamide (25 mg/kg, p.o.), (iii) huaier extract (50 mg in 100-ul normal saline, p.o.), and (iv) enzalutamide (25 mg/kg, p.o.) + huaier extract (50 mg in 100-ul normal saline, p.o.). Tumors were measured every five days and calculated using length × width^2^/2. Mice were also weighed one to two times per week to monitor for signs of drug toxicity. Tumor tissues were harvested after 25 days of treatment.

### Statistical Analysis

The results were analyzed using SPSS software (SPSS, Chicago, Illinois, USA). Student’s t-test, one-way ANOVA, and two-way ANOVA were performed. Every experiment was performed in triplicate, and differences were considered significant when p-values < 0.05.

## Results

### Huaier Extract Inhibited Prostate Cancer Cell Viability and Motility

**Figure 1:**
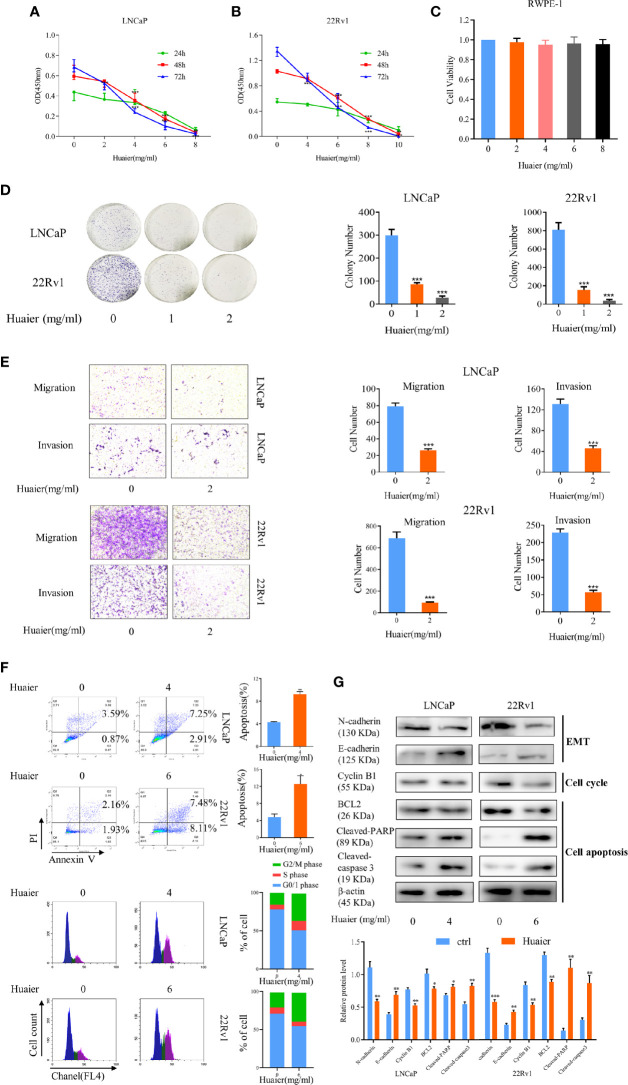
Huaier extract inhibited prostate cancer cell viability and motility. LNCaP **(A)** and 22Rv1 **(B)** cells viability were measured by cell counting kit 8 assay after treatment of huaier extract with different concentrations for 24, 48, and 72 h. **(C)** RWPE-1 cells viability was measured by cell counting kit 8 assay after treatment of huaier extract with different concentrations for 48 h. **(D)** LNCaP and 22Rv1 were treated with 0, 1, and 2 mg/ml huaier extract for clonogenic assay. Colonies were counted and representative images of colonies are shown. **(E)** LNCaP and 22Rv1 cells mobility were measured using the Transwell system as described in *Materials and Methods*. **(F)** Representative images of cell cytometry of PCa cells treated with huaier extract. **(G)** The protein levels of markers in cell apoptosis, cell cycle, and Epithelium-mesenchymal transition (EMT) were measured by western blot in PCa cells treated with huaier extract. Representative contrast-phase images are shown. *p < 0.05, **p < 0.01, ***p < 0.001.

In our study, we used two prostate cancer cell lines, LNCaP and 22Rv1, to verify the effect of huaier extract on prostate cancer. LNCaP, the hormone-sensitive prostate cancer (HSPC) cell line, expresses AR-FL, which is sensitive to AR-targeted therapy. 22Rv1, the CRPC cell line, expresses both AR-FL and AR-V7, which are resistant to AR-targeted therapy. First, to test the effect of huaier extract on PCa cells, we measured cell viability using a CCK-8 assay in which the cells were treated with different concentrations of huaier extract for different lengths of time. As shown in **Figure 1**, huaier extract markedly inhibited growth of LNCaP (**Figure 1A**) and 22Rv1 (**Figure 1B**) in time- and dose-dependent manners, but had no effects on the normal human prostatic epithelial cell line, RWPE-1 (**Figure 1C**). Moreover, Huaier extract had inhibitory effect on AR negative PCa cells (**Supplementary Figure 1**), but the inhibitory effect of huaier extract on AR-negative PCa cells were weaker than that on AR-positive PCa cells. To further examine the antitumor effects of huaier extract, a clonogenic assay was performed. As shown in **Figure 1D**, the numbers of clones were significantly decreased by huaier extract. To evaluate the effect of huaier extract on motility of LNCaP and 22Rv1, a transwell assay was performed. As shown in **Figure 1E**, huaier extract could significantly inhibit migration and invasion in both LNCaP and 22Rv1. To further explore the mechanism of inhibitory effect on cell viability, we performed flow cytometry and western blot. As shown in **Figure 1F**, huaier extract could induce PCa cells apoptosis and G2/M arrest. The data of western blot from **Figure 1G** further confirmed these results in **Figure 1F**. Collectively, these results revealed that huaier extract inhibited viability and motility of both the HSPC cell line and CRPC cell line.

### Huaier Extract Inhibited Full Length Androgen Receptor and Androgen Receptor Splicing Variant 7 Signaling Pathways

**Figure 2:**
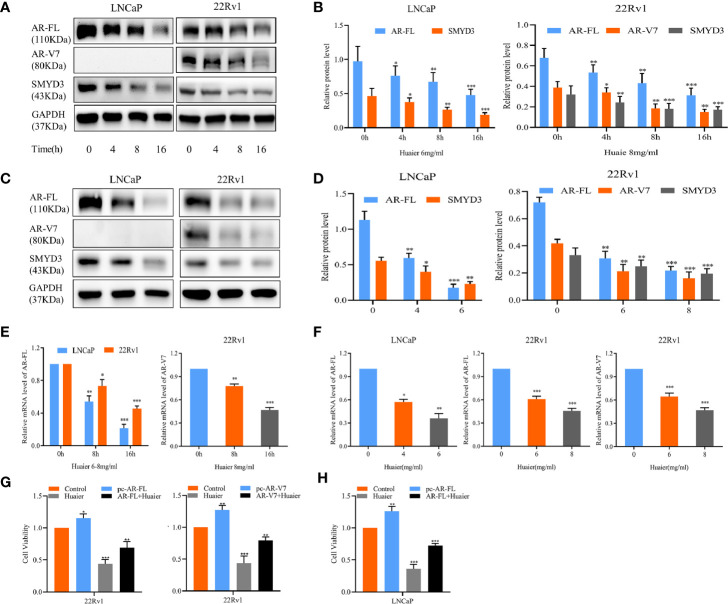
Huaier extract inhibited AR-FL and AR-V7 expression. **(A)** the protein levels were measured by western blot in LNCaP (6 mg/ml) and 22Rv1 (8 mg/ml) treated with huaier extract for 0, 4, 8, and 16 h. **(B)** Quantitative results are illustrated for panel **(A)**. **(C)** the protein levels were measured by western blot in LNCaP and 22Rv1 dose-dependently treated with huaier extract (LNCaP, 0, 4, 6 mg/ml, 22Rv1, 0, 6, 8 mg/ml). **(D)** Quantitative results are illustrated for panel **(C)**. The mRNA levels of AR-FL and AR-V7 were measured by RT-qPCR in LNCaP and 22Rv1 time-**(E)** or dose-**(F)** dependently treated with huaier extract. **(G, H)** The cell viability were measured by CCK8 in 22Rv1 and LNCaP with AR/AR-V7 overexpression upon huaier extract treatment. *p < 0.05, **p < 0.01, ***p < 0.001.

Subsequently, we considered whether the anti-viability and anti-motility effects of huaier extract corresponded with changes of AR-FL and AR-V7, as AR and related pro-oncogenic signaling play important roles in prostate carcinogenesis (3, 6, 17). We examined the protein levels of AR-FL for LNCaP and AR-V7 for 22Rv1, which were time-dependently treated with huaier extract. As showed in **Figures 2A, B**, huaier extract inhibited both AR-FL and AR-V7 expression starting at 4-h treatment in a time-dependent manner, and huaier extract also reduced the protein levels of AR-FL and AR-V7 in a dose-dependent manner as shown in **Figures 2C, D**. The above data confirmed that huaier extract could reduce protein levels of AR-FL and AR-V7, and we hypothesized that huaier extract could also affect the transcription of AR-FL and AR-V7. To test the hypothesis, we measured the mRNA levels of AR-FL and AR-V7 in LNCaP and 22Rv1 cells following treatment with huaier extract. As shown in **Figures 2E, F**, huaier extract reduced mRNA levels of AR-FL and AR-V7 in dose- and time-dependent manners. To further explore the hypothesis that huaier extract inhibit PCa growth *via* targeting AR/AR-V7, we tested huaier extract inhibitory effect through CCK8 assays after overexpression of AR/AR-V7 in PCa cells (**Supplementary Figure 2**). As shown in **Figures 2G, H**, overexpression of AR/AR-V7 could partly rescue the inhibitory effect caused by huaier extract on PCa cells. Altogether, these data demonstrated that huaier extract could suppress PCa growth *via* reducing AR-FL/AR-V7 expression in both protein and mRNA levels.

### Huaier Extract Enhanced the Protein Degradation of Full Length Androgen Receptor and Androgen Receptor Splicing Variant 7 *via* the Ubiquitin–Proteasome System Through Downregulation Ubiquitin-Specific Protease 14

**Figure 3:**
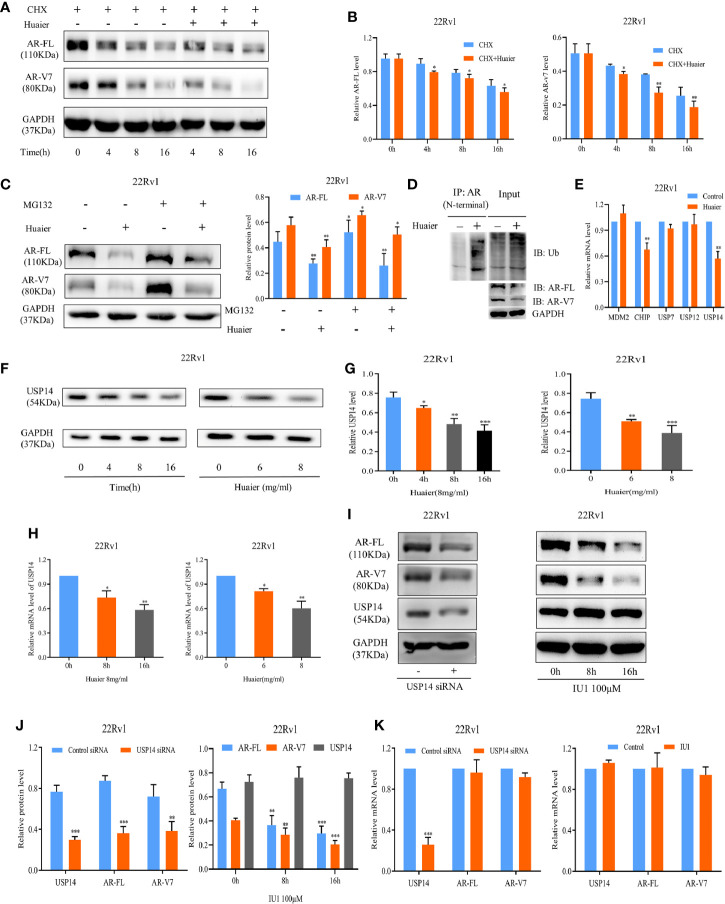
Huaier extract enhanced AR-FL/AR-V7 degradation *via* the ubiquitin–proteasome system through downregulation USP14. **(A)** the protein levels of AR-FL and AR-V7 were measured in 22Rv1 by western blot at specified time points after treatment of 50 μg/ml CHX along with or without 6 mg/ml huaier extract. **(B)** Quantitative results are illustrated for panel **(A)**. **(C)** MG132 could partly reduce huaier extract mediated inhibition of AR-FL/AR-V7 protein expression. 22Rv1 cells were treated with MG132 (10 μmol/L) in the presence or absence of 6 mg/ml huaier extract, and the protein levels of AR-FL and AR-V7 were analyzed by western blot at 16 h. **(D)** Huaier extract promoted AR-FL/AR-V7 degradation *via* the ubiquitin–proteasome system. Co-immunoprecipitation assay was performed using AR antibody (N-terminal) beads, and immunoblotted for ubiquitin (Ub) in 22Rv1 treated with 8 mg/ml huaier extract for 16 h. **(E)** The mRNA levels were measured by RT-qPCR in 22Rv1 treated with huaier extract for 16 h. **(F)** The protein levels of USP14 were measured by western blot in 22Rv1 time- and dose-dependently treated with huaier extract. **(G)** Quantitative results are illustrated for panel **(F)**. **(H)** The mRNA levels of USP14 were measured by RT-qPCR in 22Rv1 time- and dose-dependently treated with huaier extract. **(I)** The protein levels were analyzed by western blot in 22Rv1 cells transfected with USP14 small interfering RNAs (siRNAs), or treated with IU1. **(J)** Quantitative results are illustrated for panel **(I)**. **(K)** The mRNA levels were analyzed by RT-qPCR in 22Rv1 cells transfected with USP14 small interfering RNAs (siRNAs), or treated with IU1. *p < 0.05, **p < 0.01, ***p < 0.001.

There are two factors, the synthetic metabolic process of anabolism and the eliminative process of catabolism, that affect the protein levels in the cells. First, we hypothesized that AR-FL and AR-V7 might be degraded following treatment with huaier extract. To test the hypothesis, CHX, which inhibits the protein synthesis, was used with or without 6 mg/ml huaier extract. As shown in **Figures 3A, B**, co-treatment of CHX and huaier extract caused more rapid inhibition in protein levels of AR-FL and AR-V7, which meant that huaier extract could diminish the protein stability of AR-FL and AR-V7. To verify whether huaier extract induced AR-FL and AR-V7 protein degradation *via* the ubiquitin–proteasome system, MG132, the proteasome inhibitor, was added to the culture media containing huaier extract. MG132 could reduce huaier extract-mediated inhibition of AR-FL/AR-V7 protein expression (**Figure 3C**). We also measured the abundance of ubiquitinated AR using co-IP, and huaier extract could dramatically increase the level of ubiquitinated AR (**Figure 3D**).

AR protein stability is regulated by the ubiquitination and deubiquitination system. So far, the genes reported to regulate AR degradation mainly belong to two families, the E3 ubiquitin ligases such as MDM2, CHIP, and the deubiquitinases such as USP7, USP12, and USP14 (28–32). We performed a RT-qPCR screen to identify these genes’ alterations upon huaier extract treatment in 22Rv1 cells. As shown in **Figure 3E**, only USP14 was inhibited by huaier extract treatment [CHIP was also downregulated by huaier extract treatment, but it was attributed to ubiquitinated AR, resulting in degradation (29)], and we revealed huaier extract decreased USP14 in dose- and time-dependent manners (**Figures 3F–H**). We further performed siRNA-mediated knockdown assays to determine whether USP14 affects the expression of AR-FL and AR-V7, as shown in **Figures 3I–K**. Knockdown USP14 could suppress the expression of AR-FL and AR-V7 in protein levels, but no effect was seen on mRNA levels. To further verify the downregulation of AR-FL and AR-V7 *via* USP14 deubiquitinating activity, we examined the effect of IU1, a selective USP14 inhibitor that inhibits its deubiquitination function, and we found decreased AR-FL and AR-V7 in protein levels but not mRNA levels, which corresponded with results in siRNA assays. These data suggested that huaier extract induces proteasome-mediated degradation of AR-FL and AR-V7 by downregulation USP14.

### Huaier Extract Decreases the mRNA Levels of Full Length Androgen Receptor and Androgen Receptor Splicing Variant 7 Through Inhibiting SET and MYND Domain-Containing Protein 3 Signaling

**Figure 4:**
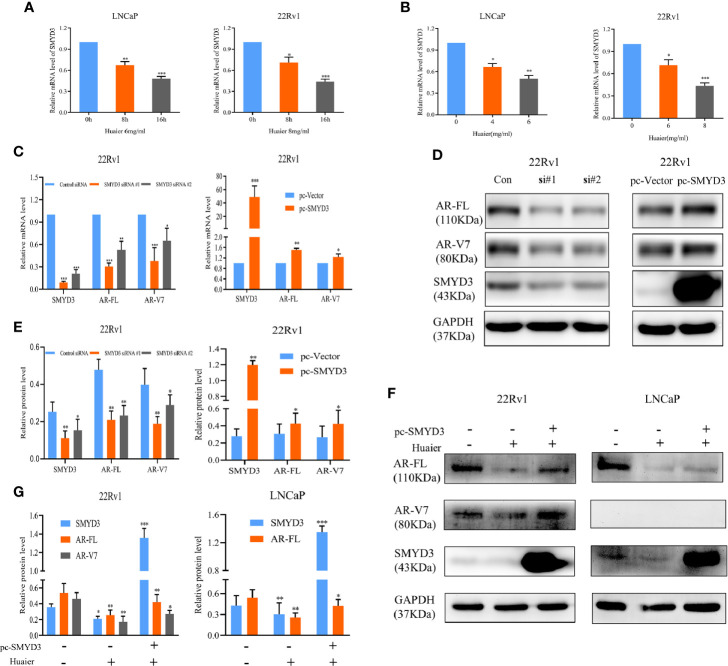
Huaier extract inhibited AR-FL and AR-V7 mRNA levels *via* targeting SMYD3 signaling. The mRNA levels of SMYD3 were measured by RT-qPCR in LNCaP and 22Rv1 time-**(A)** or dose-**(B)** dependently treated with huaier extract. The AR-FL and AR-V7 transcription **(C)** and expression **(D)** were analyzed by RT-qPCR and western blot in 22Rv1 cells transfected with SMYD3 small interfering RNAs (siRNAs), control siRNA, pc-vector, or pc-SMYD3 vector respectively. **(E)** Quantitative results are illustrated for panel **(D). (F)** 22Rv1 cells and LNCaP cells were transfected with SMYD3-cDNAs plasmid (pc-SMYD3) or control plasmid (pc-vector) for 48 h, followed by treatment of 6 mg/ml huaier extract for another 16 h, then the protein levels of AR-FL and AR-V7 were analyzed by western blot. **(G)** Quantitative results are illustrated for panel **(F)**. *p < 0.05, **p < 0.01, ***p < 0.001.

As shown in **Figures 2E, F**, huaier extract reduced mRNA levels of AR-FL/AR-V7 in a dose- and time-dependent manner. This explained the fact that co-treatment of MG132 and huaier extract did not completely rescue AR-FL and AR-V7 protein levels to that of MG132 using alone (**Figure 3C**). These results suggested that huaier extract affects AR-FL and AR-V7 transcription. We previously found that SET and MYND domain-containing protein 3 (SMYD3) can promote AR transcription (33). Therefore, we tested whether huaier extract decreased the transcription of AR-FL and AR-V7 *via* inhibiting SMYD3. First, we evaluated effects of huaier extract on SMYD3 expression *via* RT-qPCR and western blot, and we found that reduced SMYD3 both in mRNA and protein levels corresponded with alterations of AR-FL and ARV7, which also occurred in dose- and time-dependent manners (**Figures 2A, C**, and **Figures 4A, B**). Furthermore, we performed siRNA-mediated knockdown and SMYD3-plasmid-mediated overexpression assays to determine whether SMYD3 affects both the expression of AR-FL and AR-V7. As shown in **Figures 4C–E**, knockdown SMYD3 could suppress the expression of AR-FL and AR-V7, and overexpression of SMYD3 could increase the expression of AR-FL and AR-V7. To further investigate the role of SMYD3 in huaier-mediated inhibition on AR-FL and AR-V7, we first transfected plasmids of SMYD3-cDNAs to PCa cells for 48 h, and then we treated these cells with or without 6 mg/ml huaier extract for another 16 h. As shown in **Figures 4F, G**, overexpression of SMYD3 was able to partly rescue the expression of AR-FL and AR-V7. Collectively, these results revealed that huaier extract decreased the mRNA levels of AR-FL and AR-V7 by inhibiting SMYD3 signaling.

### Huaier Extract Inhibited Full Length Androgen Receptor and Androgen Receptor Splicing Variant 7 Transcriptional Activity and Nuclear Translocation

**Figure 5:**
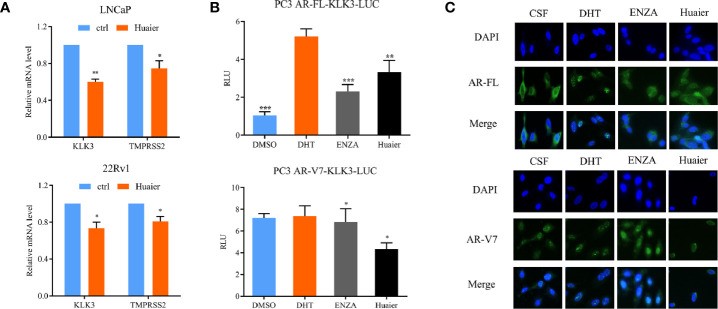
Huaier extract inhibited AR-FL and AR-V7 transcriptional activity and nuclear translocation. **(A)** the AR-FL/AR-V7 targeted genes expression were analyzed by RT-qPCR at 16 h after 6 mg/ml huaier extract treatment for LNCaP and 8 mg/ml for 22Rv1. **(B)** PC3 cells were cultured in CS-FBS condition and cotransfected with KLK3 luciferase promoter and pc-AR-FL/pc-AR-V7 for 24 h, followed by treatment with 10 nmol/L DHT, 20 μmol/L enzalutamide or 2 mg/ml huaier extract for another 24 h, and whole cell lysates were subjected to luciferase assay. **(C)** PC3 transfected with pc-AR-FL/pc-AR-V7 for 24 h in CS-FBS condition, were pre-treated with 20 μmol/L enzalutamide or 2 mg/ml huaier extract for 24 h, followed by 2 h treatment with 10 nmol/L DHT, and the cell were subjected to immunofluorescence assay as described in *Materials and Methods*. Representative images are shown. *p < 0.05, **p < 0.01, ***p < 0.001. DHT, dihydrotestosterone, ENZA, enzalutamide.

The above results confirmed that huaier extract can inhibit the expression of AR-FL and AR-V7 both in mRNA and protein levels. To further confirm the inhibition effort of huaier extract on AR-FL and AR-V7 signaling pathways, we used RT-qPCR to evaluate the expression of AR-FL/AR-V7 target genes. As shown in **Figure 5A**, huaier extract remarkably inhibited the expressions of KLK3 and TMPRSS2 in PCa cells. In order to verify the direct effect of huaier extract on the transcriptional activity of AR-FL and AR-V7, we constructed an AR-FL/AR-V7-KLK3-LUC system in AR-negative PC3 prostate cancer cells. We cotransfected KLK3 luciferase promoter with AR-FL or AR-V7 to PC3 cells in CS-FBS condition for 24 h, and cells were treated with DHT, enzalutamide, or huaier extract for another 24 h. As shown in **Figure 5B**, AR-FL could activate KLK3 promoter in the presence of DHT, whereas AR-V7 did not need DHT to activate KLK3 promoter. Enzalutamide could inhibit AR-FL activating KLK3 promoter, but not AR-V7, and what encouraged us was that huaier extract could inhibit both AR-FL and AR-V7 activations on KLK3 promoter.

To accomplish its transcriptional function on its target genes, AR must translocate to the nucleus. As we had demonstrated that huaier extract could inhibit AR-FL and AR-V7 transcriptional activity, we wondered whether huaier extract could inhibit AR-FL/AR-V7 translocation to the nucleus. To confirm that, PC3 were transfected with AR-FL or AR-V7 in CS-FBS condition for 24 h, followed by treatment with 10 nmol/L DHT, 20 μmol/L enzalutamide, or 2 mg/ml huaier extract for another 24 h, then the cells were prepared for IF assay with specific antibodies for AR-FL and AR-V7. As shown in **Figure 5C**, AR-FL translocation to the nucleus needed the stimulation of DHT, and both enzalutamide and huaier extract were able to inhibit AR-FL nuclear translocation. As for AR-V7, its accumulations in the nucleus were neither affected by the absence of DHT nor the use of enzalutamide. However, treatment with huaier extract could dramatically reduce the nuclear abundance of AR-V7.

Thus, we could conclude that the inhibition of AR-FL and AR-V7 signaling pathways by huaier extract was mainly attributable to the following three aspects. First, huaier extract could inhibit the expression of AR-FL and AR-V7 both in mRNA and protein levels; second, huaier extract was able to reduce the nuclear translocation of AR-FL and ARV7; and third, huaier extract directly inhibit AR-FL/AR-V7 transcriptional activity as transcription factors on their target genes.

### Huaier Extract Enhanced Enzalutamide Treatment

**Figure 6:**
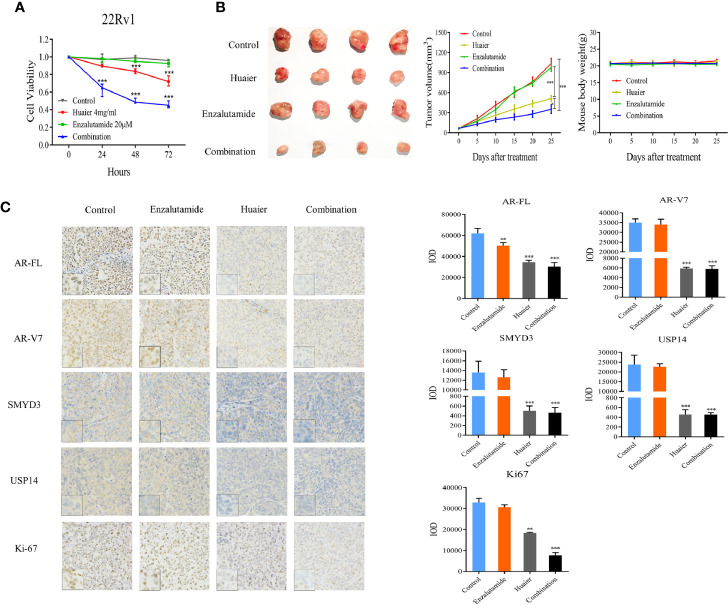
Huaier extract enhanced enzalutamide treatment. **(A)** 22Rv1 cells viability were measured by cell counting kit 8 assay after 20 μmol/L enzalutamide treatment along with or without 4 mg/ml huaier extract for 24, 48, and 72 h. **(B)** mice bearing 22Rv1 xenografts were treated with vehicle control, enzalutamide, huaier extract, or their combination for 25 days, tumor volumes were measured every five days, then the tumors were harvested. **(C)** AR-FL, AR-V7, SMYD3, USP14, and Ki67 were analyzed in tumor tissues by IHC staining and quantified as described in *Materials and Methods*. Representative images are shown. *p < 0.05, **p < 0.01, ***p < 0.001.

Enzalutamide, the drug which directly targets AR-FL, has been approved to treat docetaxel-pretreated PCa patients since 2012. However, patients treated with enzalutamide eventually acquire resistance to this agent, and an accepted reason is the presence of AR-V7 (22, 23). Our work revealed that huaier extract can inhibit AR-V7 as well as AR-FL. Thus, we hypothesized that huaier extract could enhance the effect of enzalutamide in treatment of CRPC. To verify the hypothesis, cells from the CRPC cell line 22Rv1 were treated with enzalutamide along with or without huaier extract. Then, the cell viability was examined by CCK8 assay. As shown in **Figure 6A**, 22Rv1 cells were resistant to the treatment of enzalutamide (20 μmol/L), and huaier extract (4 mg/ml) could moderately inhibit cell growth, whereas the combination of huaier extract and enzalutamide achieved a significant inhibition effect on cell viability in a time-dependent manner.

As huaier extract could enhance the treatment of enzalutamide *in vitro*, we further examined the combination effect *in vivo* using a xenograft model of 22Rv1 cells. As shown in **Figure 6B**, the xenograft models of 22Rv1 were resistant to the treatment of enzalutamide as there was no significant difference in the tumor volume to that in the control group (p > 0.05). The huaier group could inhibit tumor growth moderately, however, the combination group significantly reduced the tumor growth consistent with the results found *in vitro*, and huaier extract was safe to mouse as there was no weight change between control group and huaier extract group (**Figure 6B**). To further confirm the underlying mechanism, tumor samples of each group were analyzed by IHC for AR-FL, AR-V7, SMYD3, USP14, and Ki67. As shown in **Figure 6C**, there were no differences in IHC results between the enzalutamide group and the control group. Both the huaier group and combination group had the reduced abundance of AR-FL, AR-V7, SMYD3, and USP14 compared to the control group. However, the combination group decreased the expression of Ki-67 further than did the huaier group. These results revealed that huaier extract could overcome the resistance of enzalutamide in CRPC.

## Discussion

Traditional Chinese Medicine (TCM) has been used to treat tumors for a long time in China. With more research and high-quality clinical trials on TCM, the treatment of cancer with TCM has attracted more and more international attention and cooperation. Many studies have demonstrated that huaier extract exerted antitumor properties in a range of tumors. According to a phase IV clinical trial evaluating the benefit of huaier extract on hepatocellular carcinoma (HCC), huaier extract can improve the recurrence-free survival (RFS) rate and reduce the tumor extrahepatic recurrence rate (ERR) in HCC (26). Li et al. and Zhang et al. have demonstrated that huaier extract is effective for breast cancers (25, 34). Hu et al. have revealed that huaier extract has antitumor potential in cholangiocarcinoma (35). Xie et al. proved that huaier extract suppresses the growth of gastric cancer in their study (36). Chen et al. have revealed that huaier extract suppresses the proliferation and metastasis of lung cancer (37). Moreover, huaier extract has antitumor potential in pancreatic cancer based on a preclinical study (38). Nowadays, huaier granule, the oral medication of huaier extract, is approved by the Chinese State Food and Drug Administration for the treatment of leukaemia, osteosarcoma, malignant lymphoma, breast cancer, lung cancer, rectal cancer, liver cancer, gastric cancer, colon cancer, and pancreatic adenocarcinoma (26). However, there has thus far been no study of huaier extract antitumor effects in PCa. In our study, we found that huaier extract could inhibit the proliferation, migration, and invasion of LNCaP cells, the cell line of HSPC. As majority of new cases of PCa are localized HSPC, our data provided the evidence that huaier extract would be used for HSPC.

After a period of androgen deprivation treatment, HSPC is more commonly converted to CRPC. CRPC remains incurable, and all patients with CRPC eventually acquire resistance to the second generation of hormonal drug: enzalutamide (22). In our study, we found that huaier extract had antiproliferative effects on 22Rv1, a CRPC cell line. These results revealed that huaier extract consistently has antitumor effects either in the HSPC stage or the CRPC stage. AR plays a pivotal role in prostatic carcinogenesis, and AR related signaling pathways retain the tumor characteristics of HSPC (3–7). Moreover, AR related transcriptional activity is also the main oncogenic signaling in CRPC, particularly the appearance of AR-V7 (2, 10). It is well known that AR-V7 is a biomarker of androgen receptor signaling inhibitor resistance in CRPC (20–23). Importantly, recent studied have demonstrated that patients harboring AR-V7 are resistant to enzalutamide treatment (22). Worse still, there is no efficacious drug targeting AR-V7. In our study, we found that huaier extract significantly inhibited the expressions of AR-FL and AR-V7, as well as their transcriptional activity and the nuclear translocation. Moreover, our study confirmed that huaier extract can re-sensitize enzalutamide-resistant prostate cancer cells to enzalutamide treatment *in vitro* and *in vivo* models. Our data also provided the evidence that huaier extract would be used for CRPC.

pt?>To further explore the underlying mechanisms that huaier extract inhibit the expression of AR-FL and AR-V7, we focused our attention on the modulators that could regulate AR transcription and post-translational modification (PTM). SMYD3, functioning as methyltransferase, plays a pivotal role in methylation of targeted proteins (39). Many studies have demonstrated that SMYD3 acts as an oncogene in hepatocellular carcinoma, breast cancer, ovarian cancer, and renal cell carcinoma, in which SMYD3 regulates downstream targets involved in proliferation and metastasis (40–43). Lobo et al. found that SMYD3 acted as the prognostic biomarker in PCa, and patients with a higher expression of SMYD3 always got the worse disease-specific survival (DSS) (44). Furthermore, our previous study confirmed that the increased expression of SMYD3 was the oncogenic driver in PCa by stimulation of AR transcription (33). In our study, we confirmed that SMYD3 can also regulate the transcription of AR-V7 in PCa cells, and the effects of huaier extract on AR-FL/AR-V7 were partly mediated by downregulating SMYD3 in PCa. To our knowledge, this is the first study demonstrating that SMYD3 could regulate the expression of AR-V7. Our results may mechanistically illustrate the phenomenon that SMYD3 prefers to highly express in the more aggressive PCa (45).

PTM is responsible for the signal transduction of phosphate, ubiquitin, methyl, acetyl, and glycosyl groups from one protein to another (46, 47). Many studies revealed that PTM is involved in tumor occurrence and progression, including prostate cancer (48). PTM of AR has been a subject of considerable interest, as it could modify AR transcriptional activity, nuclear translocation, and protein stability (49, 50). Among the PTMs occurring in AR, ubiquitination of AR has gotten more attention from researchers, as it plays an important role in AR stability. Recent studies have revealed that MDM2, CHIP, USP7, USP12, and USP14 are the regulators in ubiquitination and deubiquitination of AR (28–32). In our study, we confirmed that huaier extract enhanced proteasome-mediated protein degradation of AR-FL and AR-V7 by downregulating USP14. The data that USP14 regulated AR-V7 in our study provided new insights in USP14 function, and it corresponds with the conclusion that inhibition of USP14 can enhance enzalutamide treatment in hormone-sensitive cancer cells (51). Our results were consistent with that ubiquitin-proteasome degradation is a common method for AR proteolysis in PCa (28). However, recent study proved that autophagy serves as an alternative way for AR degradation (52). 3-MA, the autophagy inhibitor can increase AR/AR-V7 protein stability in 22Rv1 (53). Huaier extract was proved to attenuate the activation of PI3K/AKT/mTOR pathway, which is classically related to autophagy initiation (27, 54). Therefore, it is reasonable to hypothesize that huaier extract can promote AR/AR-V7 degradation *via* activating autophagy in PCa cells, which need to be explored in the future.

**Figure 7:**
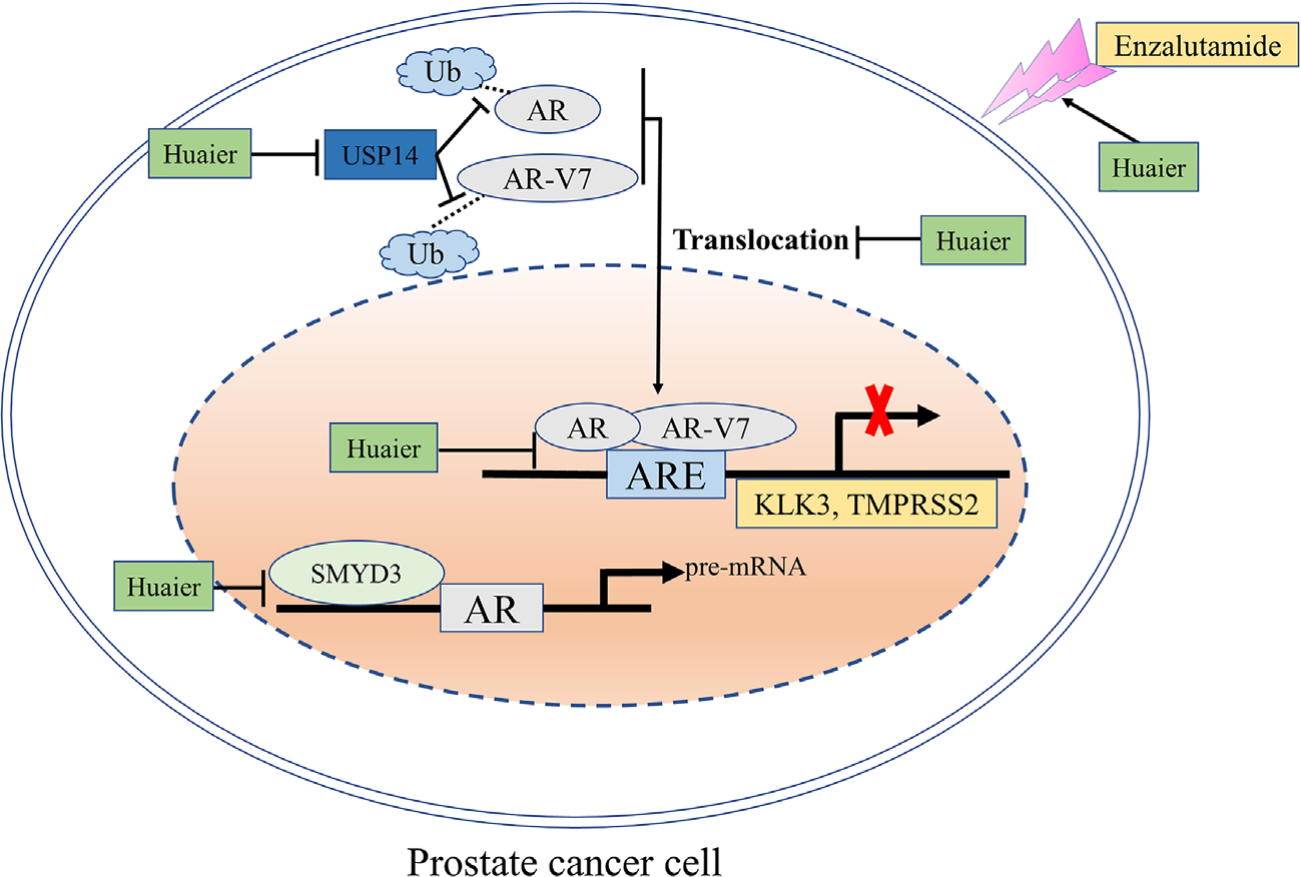
A schematic model: the mechanisms through which huaier extract inhibits the growth of prostate cancer. The effects of huaier extract in prostate cancer shown in this study are: (1) down-regulation the expression of AR-FL/AR-V7 both in mRNA and protein levels *via* targeting SMYD3 signal pathway and USP14 related ubiquitin–proteasome system; (2) reducing the nuclear translocation of AR-FL as well as the nuclear accumulation of AR-V7, which, in turn, inhibits their transcriptional activity as transcription factors to their target genes; (3) enhancing enzalutamide treatment.

In summary, our study revealed that huaier extract is effective in both hormone-sensitive and castration-resistant prostate cancer *via* targeting AR-FL and AR-V7 signaling pathways (**Figure 7**). Furthermore, huaier extract exhibits a synergistic effect with enzalutamide and reverses its resistance. All these results indicate that Huaier extract may be a potential promising therapeutic drug for patients with PCa.

## Data Availability Statement

The original contributions presented in the study are included in the article/**Supplementary Material**. Further inquiries can be directed to the corresponding authors.

## Ethics Statement

The animal study was reviewed and approved by the Ethics Committee on Scientific Research of Shandong University, Qilu Hospital.

## Author Contributions

YF and ZF designed and supervised the study. ZL carried out the experiments and drafted the manuscript. JL and KY technically supported and revised of the manuscript. ZL and CL analyzed the experimental results. All authors contributed to the article and approved the submitted version.

## Funding

This study was supported by the National Natural Science Foundation of China (Nos. 81572515, 81472395, and 81672522) and the National Science Foundation for Young Scientists of Shandong (ZR2020QH245).

## Conflict of Interest

The authors declare that the research was conducted in the absence of any commercial or financial relationships that could be construed as a potential conflict of interest.
