# Supplementary material for: Huaier Extract Inhibits Prostate Cancer Growth via Targeting AR/AR-V7 Pathway
Source: Front Oncol. 2021 Feb 23;11:615568. doi: 10.3389/fonc.2021.615568 (PMC7940541; doi:10.3389/fonc.2021.615568)
Supplement: Supplementary file 1 [file DataSheet_1.docx]

**Table 1.** The chemical composition of proteoglycan (protein-bound polysaccharides) extracted from Huaier

| Composition of Amino acid (1) | |  | Composition of Polysaccharide (2, 3) | |
| --- | --- | --- | --- | --- |
| amino acid | percent% |  | monosaccharide | molar ratio |
| Asp | 1.418 |  | L-Fucose | 0.51 |
| Thr | 0.731 |  | L-Arabinose | 1.15 |
| Ser | 0.626 |  | D-xylose | 1.48 |
| Glu | 3.525 |  | D-mannose | 1.39 |
| Pro | 0.740 |  | D-galactose | 1 |
| Gly | 1.073 |  | D-Glucose | 3.24 |
| Ala | 0.662 |  |  |  |
| Cys | 0.160 |  |  |  |
| Val | 0.652 |  |  |  |
| Met | 0.120 |  |  |  |
| Ile | 0.398 |  |  |  |
| Leu | 0.569 |  |  |  |
| Tyr | 0.249 |  |  |  |
| Phe | 0.347 |  |  |  |
| Lys | 0.689 |  |  |  |
| His | 0.267 |  |  |  |
| Try | 0.097 |  |  |  |
| Arg | 0.645 |  |  |  |
| total | 12.93 |  |  |  |

1. Guo Y, Cheng P, Chen, Y, Zhou X. Isolation and analysis of the polysaccharide of Huaier mycelium. *Chinese J Biochem Pharm*. (1993) 63:56-9. (in Chinese)

2. Guo Y, Cheng P, Chen Y, Zhou X, Yu P, Li Y, et al. Studies on the Constituents of Polysaccharide from the Hyphae of Trametes Robiniophila(II)——Identification of Polysaccharide from the Hyphae of Trametes Robiniophila and Determination of Its Molar Ratio. *J Chinese Pharm U*. (1992) 23:155-7. (in Chinese)

3. Wang Y, Liu Y, Hu Y, Optimization of polysaccharides extraction from Trametes robiniophila and its antioxidant activities. *Carbohydr Polym*. (2014) 111:324–32.

**Table 2.** Nucleotide sequence of primers used for RT-qPCR

Gene name Oligonucleotide sequence (5'->3')

| AR-FL | Forward GGT GAG CAG AGT GCC CTA TC |
| --- | --- |
|  | Reverse GAA GAC CTT GCA GCT TCC AC |
| AR-V7 | Forward AAC AGA AGT ACC TGT GCG CC |
|  | Reverse TCA GGG TCT GGT CAT TTT GA |
| KLK3 | Forward AGG CCT TCC CTG TAC ACC AA |
|  | Reverse GTC TTG GCC TGG TCA TTT CC |
| TMPRSS2 | Forward CTG CCA AGG TGC TTC TCA TT |
|  | Reverse CTG TCA CCC TGG CAA GAA TC |
| SMYD3 | Forward TGC TGA TGA CCA GTG AGG AGC G |
|  | Reverse AAC CTG CTC CCA CTT CCA GTG T |
| MDM2 | Forward GCCCTTCGTGAGAATTGGCT |
|  | Reverse AAGCCCTCTTCAGCTTGTGTT |
| CHIP | Forward CAACCGGGCCTTGTGCTAC |
|  | Reverse GGCCTCATCATAGCTCTCCA |
| USP7 | Forward CCAACCGAGGGGGATGATTC |
|  | Reverse TGGGTATGGTGCCCTCTACA |
| USP12 | Forward TGTGAAGAGTGTCGCAGCAA |
|  | Reverse CCACAACAGCAACAAGGTCGT |
| USP14 | Forward GCCGCTCTACTCCGTTACTG |
|  | Reverse AACTTTCTGTCTGGCAGGCT |
| β-Actin | Forward TGA CGT GGA CAT CCG CAA AG |
|  | Reverse CTG GAA GGT GGA CAG CGA GG |
