# Supplementary material for: Huaier Extract Inhibits Prostate Cancer Growth via Targeting AR/AR-V7 Pathway
Source: Front Oncol. 2021 Feb 23;11:615568. doi: 10.3389/fonc.2021.615568 (PMC7940541; doi:10.3389/fonc.2021.615568)

**Supplementary Fig. 1.** The inhibitory effect of huaier extract on AR-negative PCa cells.

PC3 (**A**) and DU145 **(B**) cells viability were measured by cell counting kit

8 assays after treatment of huaier extract with different concentrations for 24, 48, 72

hours.

**Supplementary Fig. 2.** Overexpression of AR/AR-V7 in PCa cells. The mRNA levels

of AR/AR-V7 were measured by qPCR in 22Rv1 **(A)** and LNCaP **(B)** cells transfected

with AR-FL or AR-V7 cDNAs plasmid.

**Supplementary Fig. 1.**


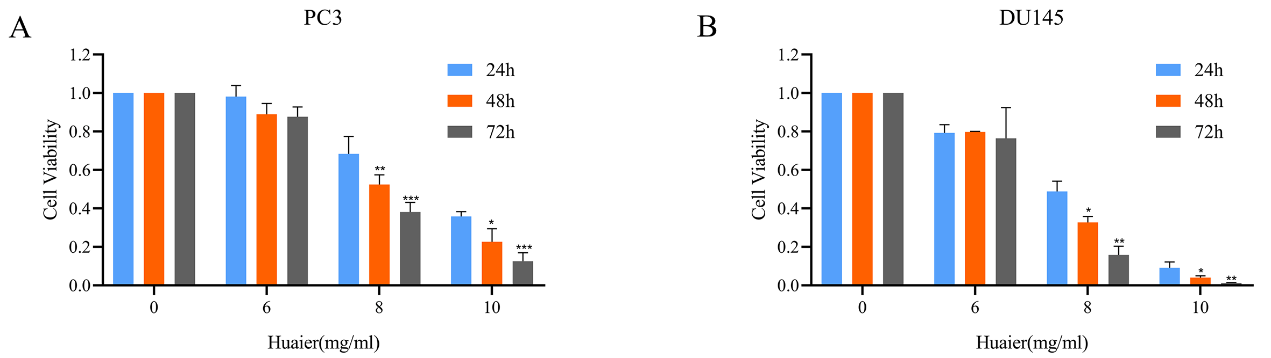


**Supplementary Fig. 2.**


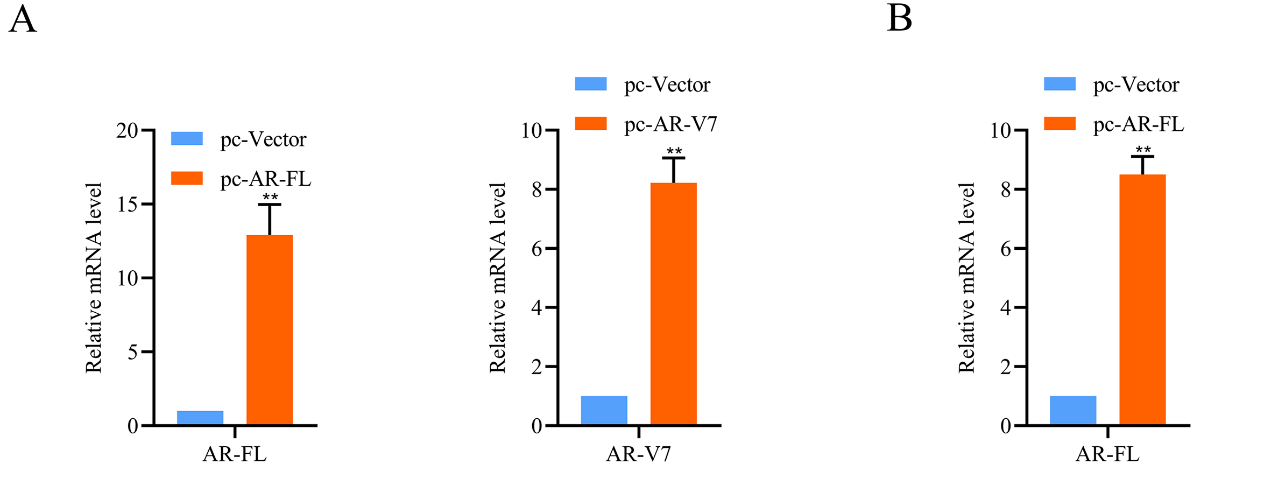

Supplement: Supplementary file 2 [file DataSheet_2.docx]
